# Evaluation of COVID-19 Booster Vaccine Effectiveness

**DOI:** 10.3390/v17020179

**Published:** 2025-01-26

**Authors:** Bingqian Qu, Dingmei Zhang

**Affiliations:** 1 Department of Infectious Diseases, University Hospital Heidelberg, 69120 Heidelberg, Germany; 2 European Virus Bioinformatics Center, 07743 Jena, Germany; 3 Department of Epidemiology, School of Public Health, Sun Yat-sen University, Guangzhou 510080, China

## 1. Introduction

The COVID-19 pandemic resulted in at least 776 million confirmed cases and 7 million deaths worldwide by the end of 2024. Substantial public support and unprecedented scientific collaboration enabled the swift development and production of COVID-19 vaccines in record time. Since 2021, more than 13.3 billion vaccine doses have been administered, preventing over half of COVID-19-related hospitalizations and severe outcomes, including death [1]. Thanks to these efforts, the emergency phase of COVID-19 ended in May 2023. However, severe acute respiratory syndrome coronavirus 2 (SARS-CoV-2), as a zoonotic virus, continues to evolve, spread globally, and endanger vulnerable populations, particularly older adults, immunocompromised individuals, and those with comorbidities. Therefore, the World Health Organization recommends one dose of (re)vaccination for all adults, adolescents, and children and two or three doses for vulnerable groups and healthcare workers.

The first approved vaccines were developed based on the SARS-CoV-2 ancestral strain [2]. Due to breakthrough infections caused by antigenic drift and the neutralization escape of emerging variants of concern (VOCs), adapted vaccines were created to protect against these variants following initial or booster vaccination [3]. Currently, two messenger RNA (mRNA) vaccines i.e., Comirnaty (BNT162b2, Pfizer/BioNTech, BioNTech, Mainz, Germany) and Spikevax (mRNA-1273, Moderna, Cambridge, Massachusetts, USA), as well as one virus-like particle vaccine, i.e., Nuvaxovid (NVX-CoV2373, Novavax, Gaithersburg, Maryland, USA), have been approved by the U.S. Food and Drug Administration for preventing infections caused by Omicron variants (lineages XBB.1.5, JN.1, and KP.2). Additionally, a recombinant protein-based vaccine, Bimervax (HIPRA Human Health, Amer, Catalonia, Spain), has been authorized by the European Medicines Agency as a booster dose for individuals previously vaccinated with an mRNA vaccine.

In real-world settings, hybrid immunity is an immune protection conferred by a combination of SARS-CoV-2 infection and vaccination. Immunization with mRNA-encoded or recombinant spike protein subunit vaccines induces spike antigen-specific neutralizing antibodies (nAbs) and memory B and T cell responses. Moreover, natural infection elicits robust humoral and adaptive immunity, thereby preventing viral dissemination and facilitating viral clearance. Individuals with hybrid immunity exhibit the highest magnitude of antiviral immunity and the longest durability of protection [4]. Three consecutive antigen exposures—either through three doses of vaccination or a breakthrough infection in twice-vaccinated individuals—result in increased anti-spike antibody avidity and more potent neutralizing activity against VOCs, including Omicron variants [5].

Two or three doses of vaccination may provide the most intense immune responses and the broadest protection against Omicron VOCs. One or two boosters of mRNA vaccines increase the half-life of serum nAb titers against the ancestral strain to several months. However, the neutralization capacity against Omicron VOCs (BA.2.75, BQ.1.1, and XBB.1.5) relative to the ancestral strain declines dramatically in titers. NAb titers against the newer VOCs become undetectable in almost all vaccinees within six months, although antigen-specific memory B and T cells persist longer [6].

Immune imprinting refers to the influence of prior vaccination or natural infection on future antibody response patterns when the host is exposed to distinct VOC antigens. Upon successive exposure to a heterologous VOC, the previous antibody pool could be recalled, but this would compromise the response to VOC-based boosters, as non-nAbs or low-affinity binding antibodies (bAbs) to the VOC provide limited or no protection [7]. Repeated exposure to Omicron variants by receiving two up-to-date mRNA vaccine boosters can mitigate previous vaccination-induced immune imprinting and elicit broad nAb responses in plasma and nasal mucosa in humans [8].

In this Special Issue, we have presented eight research papers evaluating COVID-19 booster vaccine effectiveness (VE). These papers address topics on the real-world VE of booster doses, immunogenicity and antibody responses, VE against emerging VOCs, and recommendations for special populations.

## 2. Real-World Vaccine Effectiveness of Booster Doses

The safety and efficacy of COVID-19 vaccines have been evaluated in large real-world cohorts. For instance, Brazil experienced four waves of COVID-19 caused by VOCs (lineages B.1, P.1, B.1.1.529, and BA.4/BA.5). Hojo-Souza et al. analyzed hospitalized Brazilian COVID-19 patients using the largest Brazilian database (n = 633,820). Four in-hospital parameters of severe outcomes were assessed as relative risks. Their analysis demonstrated that individuals who received booster vaccinations had lower risks of invasive ventilation requirements and in-hospital death in all age groups, compared to unvaccinated or not fully vaccinated patients [9]. This underscores the significance of booster vaccination in reducing adverse outcomes for hospitalized patients on a national scale.

## 3. Immunogenicity and Antibody Responses

Understanding sequential immunization with homologous and heterologous boosters has helped inform vaccination strategies that are able to maximize protective efficacy and mitigate waning immunity. Nah et al. investigated antibody response and immunogenic durability following a booster dose of Comirnaty in homologous (Comirnaty/Comirnaty, Spikevax/Spikevax, Vaxzevria/Vaxzevria) and heterologous (Vaxzevria/Comirnaty) two-dose vaccination regimens among healthcare workers in South Korea (*n* = 869). One month after the booster dose, neutralizing antibody (nAb) levels were significantly higher in homologous mRNA-vaccine groups than in the Vaxzevria/Vaxzevria and Vaxzevria/Comiranty groups. Despite waning antibody titers within three to ten months, the Comirnaty booster dose elicited a robust humoral response across various vaccination schemes, with a more pronounced effect in homologous mRNA vaccine recipients [10]. Addressing waning immunity remains a critical consideration for future vaccine development.

Ventura-Enríquez et al., on the other hand, studied the effects of a Vaxzevria (AstraZeneca, Cambridge, England) booster dose following two doses of Comirnaty among navy personnel in Mexico (*n* = 392). Anti-spike bAb levels were lower in uninfected individuals than in those with prior COVID-19 infections, but these levels significantly increased nine months post-booster vaccination in the participants, regardless of natural infection status [11]. Although the marketing authorization for Vaxzevria has been withdrawn, further studies are needed to evaluate the immunogenicity of Bimervax.

Currently, most individuals are no longer naïve to SARS-CoV-2 due to prior infection and/or vaccination. A clinical study by Wang et al. characterized the dynamics of antibody responses nine months post-vaccination in individuals exposed to SARS-CoV-2 infection (*n* = 58) and healthy donors in China (*n* = 25). NAbs against ancestral or Omicron strains were detectable in previously infected individuals even one year post-infection. Receiving inactivated vaccines further enhanced antibody levels in infected individuals, which were significantly higher than in healthy individuals given a booster dose. In infected individuals, nAb levels plateaued six months post-vaccination but declined constantly in healthy controls [12]. These findings suggest that natural infection may elicit more sustained and cross-reactive immunological protection against VOCs by targeting diversely conserved epitopes in viral membranes, nucleocapsids, and other antigens.

## 4. Vaccine Effectiveness Against Emerging Variants of Concern

Omicron variants (e.g., XBB.1.5, JN.1, and KP.2) remain predominant and continue to evolve. Fernández-Ciriza et al. conducted a prospective study to assess the breadth and durability of antibody responses in a cohort of healthcare workers in Spain (*n* = 678). Participants were categorized by their history of primo- or reinfection and primary vaccination schedules. Individuals with primoinfection by Omicron variants showed higher antibody titers than those reinfected after six months of receiving a booster dose of mRNA vaccines [13]. Primoinfection with the ancestral strain or an early VOC appears to dampen booster VE, as immune imprinting recalls cross-reactive memory B cells but produces fewer Omicron-specific B cells.

Two research groups investigated the VE of booster doses in patients with kidney transplants or B cell non-Hodgkin’s lymphoma (B-NHL). When timed appropriately, the booster effect in lymphoma patients could be comparable to that in healthy individuals [14]. After a booster dose, nAb titers and CD4^+^ T cell responses to Omicron spike peptides were low in both kidney transplant recipients (KTRs) and healthy controls. Notably, when CD4^+^ T cells were challenged with ancestral spike peptides, reactivity was observed, whereas Omicron spike peptides elicited weaker responses in both groups [15].

Predictive models based on nAb titers could estimate VE against emerging VOCs. Gardner and Kilpatrick developed a model that predicts VE using VOC- and vaccine-specific nAb titer ratios (NATR_tot_) for symptomatic disease and hospitalization across a range of mean nAb titers [16]. Such models in corporations with key immunological markers could guide the selection of new VOCs, study immune evasion, and support the design of adapted vaccines.

## 5. Recommendations for Special Populations

Special populations, including the elderly, infants, pregnant women, and immunocompromised or immunodeficient individuals, require tailored vaccination strategies due to heightened risks and potential immune escape. Saito et al. examined the VE of a first booster vaccination in B-NHL patients (*n* = 186). The study confirmed that booster effectiveness was largely influenced by the timing of the last anti-cancer treatment. The VE in B-NHL patients was comparable to healthy controls if more than one year had passed since their last treatment. Otherwise, the VE was significantly poorer when treatment occurred within the preceding 12 months. Approximately 10% of B-NHL patients receiving a booster dose experienced breakthrough infections, most of which were mild [14]. This highlights that anti-CD20 monoclonal antibodies impair memory B cells, and recovery from B cell depletion takes roughly one year. Consequently, booster vaccination is not recommended during active immunosuppressive treatment.

Conversely, KTRs require long-term immunosuppressive therapy (e.g., corticosteroids, cyclosporine A, mycophenolate, and tacrolimus). After a booster dose, nAbs titers in KTRs (*n* = 7) were lower than in healthy controls (*n* = 8) and were less effective against Omicron variants [15].

## 6. Concluding Remarks

This Special Issue has explored multiple aspects of COVID-19 booster vaccines, from VE against new VOCs to recommendations for special populations. While updated vaccines have proven effective in reducing severe illness, areas for improvement remain. Given the length limitation of this editorial, below we briefly outline challenges in developing next-generation vaccines and propose possible solutions:

(1) Understanding long-term safety and immune responses requires extended observation periods. Current vaccines provide only time-limited immunity, with waning efficacy necessitating booster doses. Addressing this technical bottleneck is essential.
(2) Most approved vaccines target the rapidly evolving spike protein, which undergoes antigenic drift. Developing universal coronavirus or pan-sarbecovirus vaccines that target conserved viral components is crucial for broad-spectrum protection.
(3) Injectable vaccines provide inadequate protection at respiratory mucosal surfaces and constrain their ability to prevent transmission. Overcoming these challenges will require the continuous monitoring of SARS-CoV-2 evolution and advancements in vaccine design, such as the development of nasal vaccines.
